# Tuning Green to Red Color in Erbium Niobate Micro- and Nanoparticles

**DOI:** 10.3390/nano11030660

**Published:** 2021-03-08

**Authors:** Susana Devesa, Joana Rodrigues, Sílvia Soreto Teixeira, Aidan P. Rooney, Manuel P. F. Graça, David Cooper, Teresa Monteiro, Luís C. Costa

**Affiliations:** 1 Centre for Physics University of Coimbra (CFisUC), Physics Department, University of Coimbra, Rua Larga, 3004-516 Coimbra, Portugal; 2 i3N and Physics Department, University of Aveiro, 3810-193 Aveiro, Portugal; silvia.soreto@ua.pt (S.S.T.); mpfg@ua.pt (M.P.F.G.); 3 CEA LETI-Minatec, 17 Rue des Martyrs, 38054 Grenoble CEDEX 9, France; aidan.rooney@cea.fr (A.P.R.); david.cooper@cea.fr (D.C.)

**Keywords:** erbium niobate, sol–gel, PL, PLE, TRPL

## Abstract

Tetragonal Er_0.5_Nb_0.5_O_2_ and monoclinic ErNbO_4_ micro- and nanoparticles were prepared by the citrate sol–gel method and heat-treated at temperatures between 700 and 1600 °C. ErNbO_4_ revealed a spherical-shaped crystallite, whose size increased with heat treatment temperatures. To assess their optical properties at room temperature (RT), a thorough spectroscopic study was conducted. RT photoluminescence (PL) spectroscopy revealed that Er^3+^ optical activation was achieved in all samples. The photoluminescence spectra show the green/yellow ^2^H_11/2_, ^4^S_3/2_→^4^I_15/2_ and red ^4^F_9/2_→^4^I_15/2_ intraionic transitions as the main visible recombination, with the number of the crystal field splitting Er^3+^ multiplets reflecting the ion site symmetry in the crystalline phases. PL excitation allows the identification of Er^3+^ high-energy excited multiplets as the preferential population paths of the emitting levels. Independently of the crystalline structure, the intensity ratio between the green/yellow and red intraionic transitions was found to be strongly sensitive to the excitation energy. After pumping the samples with a resonant excitation into the ^4^G_11/2_ excited multiplet, a green/yellow transition stronger than the red one was observed, whereas the reverse occurred for higher excitation photon energies. Thus, a controllable selective excited tunable green to red color was achieved, which endows new opportunities for photonic and optoelectronic applications.

## 1. Introduction

Rare-earth (RE) niobates, such as orthoniobates RENbO_4_, are expected to have interesting luminescence properties related to the lanthanide elements in their lattice [1,2,3,4]. By changing the RE ion in the niobate host, it is possible to change and even control the spectral range of the emission, resulting in tunable phosphors for photonic and optoelectronic applications [1]. As such, RENbO_4_ have been recognized as efficient luminescent materials [1,2]. Indeed, these materials exhibit low phonon cut-off energies, which make them suitable for optical applications, as the transition probability of the intra-4f^n^ is enhanced in such case, also presenting long emission lifetimes [2,5]. Several RE ions have been employed in the formation of RE niobate materials (RE = La, Ce, Pr, Nd, Sm, Eu, Dy, Ho, and Er) [1,3,6,7,8,9,10] and their luminescence properties have been widely investigated. Among those, erbium niobates possess interesting properties for optical applications owing to the trivalent Er ion (Er^3+^) characteristics [11,12]. In the trivalent charge state, Er^3+^ has a free ion 4*f*^11^ electronic configuration, with a ^2S+1^L_J_ ground state corresponding to the ^4^I_15/2_ multiplet [13,14], and presents sharp atomic-like transitions in the visible and near-infrared spectral regions [15]. Concerning the visible region, the main optical transitions are in the green and red spectral ranges, corresponding to the ^4^S_3/2_→^4^I_15/2_ and ^4^F_9/2_→^4^I_15/2_ transitions, respectively. In the present work, we demonstrate that erbium niobate micro- and nanoparticles prepared by the citrate sol–gel method, with subsequent heat treatment at temperatures between 700 and 1600 °C, exhibit sharp emission lines due to those intraionic transitions, and that a controllable room temperature (RT) tunable green to red color can be achieved by varying the excitation energy.

## 2. Materials and Methods

### 2.1. Erbium Niobate Preparation

Polycrystalline erbium niobate powders were prepared by the citrate sol–gel method. For this, stoichiometric amounts of high-purity Er(NO_3_)_3_·5H_2_O (Aldrich) and NbCl_5_ (Merck) were dissolved in a minor amount of hydrogen peroxide (3% V/V). Subsequently, the starting materials were dispersed in a combination of citric acid and ethylene glycol solutions, in a molar ratio of 1:3, employed as chelating agent and reaction medium, respectively. The final mixture was continuously stirred for 7 days until a clear colloidal suspension was formed. After that, the resulting suspension was dried at 500 °C for 6 h to evaporate the solvent. Finally, the obtained powders were heat-treated (HT) at 700, 800, 900, 1100, 1200, 1400, 1500, and 1600 °C for 4 h, with a heating rate of 5 °C/min. Hereafter, the samples will be designated as HT followed by the corresponding treatment temperature, e.g., HT1600 for the sample treated at 1600 °C. 

### 2.2. Structural and Morphological Characterization

Thermal measurements were carried out by Hitachi STA7300 equipment (Hitachi, Woodland, USA), in nitrogen atmosphere, in a temperature range from RT to 1500 °C, with a heating rate of 5 °C/min.

The X-ray diffraction (XRD) data were collected in an Empyrean diffractometer (CuKα radiation, λ = 1.54060 Å, PANalytical XPert-Pro, Almelo, The Netherlands) at 45 kV and 40 mA, with a curved graphite monochromator, an automatic divergence slit, a progressive receiving slit and a flat plane sample holder in a Bragg–Brentano parafocusing optics configuration. Intensity data were collected by the step counting method (step 0.02° in 1 s) in the 2θ angle range of 10°–70°.

In order to confirm the crystal structure and acquire additional structural information on the samples with pure ErNbO_4_, Rietveld refinement was carried out.

The morphology of the samples was analyzed by scanning electron microscopy (SEM). The images were obtained on a TESCAN-Vega III instrument (Kohoutovice, Czech Republic). Prior to the microscopic observation, the samples were covered with carbon. For a selected sample (the one HT at the highest temperature), a transmission electron microscope (TEM) specimen was prepared by drop casting a suspension of ground powder and ethanol onto a holey carbon TEM grid. Scanning transmission electron microscopy (STEM) characterization was performed in a probe-side aberration-corrected FEI Titan Themis S/TEM (FEI Company, Eindhoven, The Netherlands) with a 200 kV 150 pA electron beam. Both bright field and high-angle annular dark field (HAADF) detectors were used to identify the particles as crystalline and characterize their morphology and size. Energy-dispersive X-ray (EDX) spectrum imaging was acquired in parallel. The relative intensities of the erbium, niobium, and oxygen characteristic edges were used to map the distribution of these elements across the particles.

Micro-Raman spectroscopy measurements on the heat-treated samples in air were conducted in a Horiba Jobin Yvon HR800 spectrometer (Horiba Scientific, Kyoto, Japan) in backscattering configuration by exciting the samples with a 442 nm line from a cw He-Cd laser (Kimmon IK Series, Fukushima, Japan) and focusing with an objective of 50× magnification.

### 2.3. Optical Characterization

All samples were analyzed by steady-state macro-photoluminescence (PL) spectroscopy at RT. For the PL analysis, two different instruments and excitation sources were employed. Firstly, the samples were assessed in a Fluorolog-3 Horiba Scientific set-up (Horiba Scientific, Kyoto, Japan) with a double additive grating Gemini 180 monochromator (1200 groves/mm and 2 × 180 mm) in the excitation and a triple grating iHR550 spectrometer (Horiba Scientific, Kyoto, Japan) in the emission (1200 grooves/mm and 550 mm), using a 450 W Xe lamp as the excitation source. The same equipment was used for PL excitation (PLE) measurements. The PLE was acquired by setting the monochromator to the maxima of the Er^3+^ visible intraionic lines, and the excitation was then scanned towards higher energies. Additionally, the samples were further excited with the 325 nm line of the He-Cd laser (power density I_0_ < 0.6 W/cm^2^). The luminescence radiation was dispersed by a SPEX 1704 monochromator (1 m, 1200 grooves/mm, (Horiba Scientific, Kyoto, Japan)) and detected with a cooled Hamamatsu R928 photomultiplier (Hamamatsu, North Coast, Stanford, USA). RT time-resolved PL (TRPL) spectra were acquired in the same Fluorolog-3 system using a pulsed Xe lamp (operating at up to 25 Hz) and the excitation was fixed either at 300 or 379 nm, which corresponds to the excitation into the high electronic excited levels of Er^3+^. The TRPL signal was collected by setting a sample window of 0.2 ms, with 61 ms of time per flash and a flash count of 200. Time delays were varied between 0.05 and 5 ms after flash.

## 3. Results and Discussion

### 3.1. Thermal and Structural Analysis

**Figure 1:**
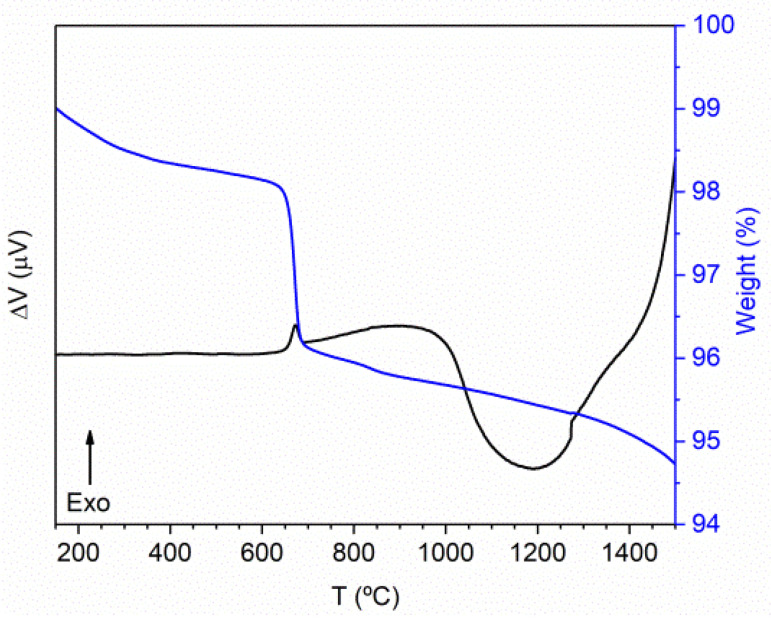
DTA/TGA thermograms of the Er-Nb obtained powders.

Figure 1 shows the results from differential thermal analysis (DTA) and thermogravimetric analysis (TGA), allowing the evaluation of the temperatures for which crystalline phases can be formed. An exothermic phenomenon, related to the formation of a crystalline structure, centered at 670 °C, and a wide endothermic one, centered at around 1200 °C, was measured. This thermal analysis was conducted up to 1500 °C, which is the maximum temperature of the equipment, with a total weight loss of around 5.3%. The thermograms suggest that for temperatures above 1500 °C, due to the increasing ∆V, an exothermic phenomenon should probably exist before the melting point. Based on these results, the selected temperatures for the heat treatments were chosen between 700 and 1600 °C.

**Figure 2:**
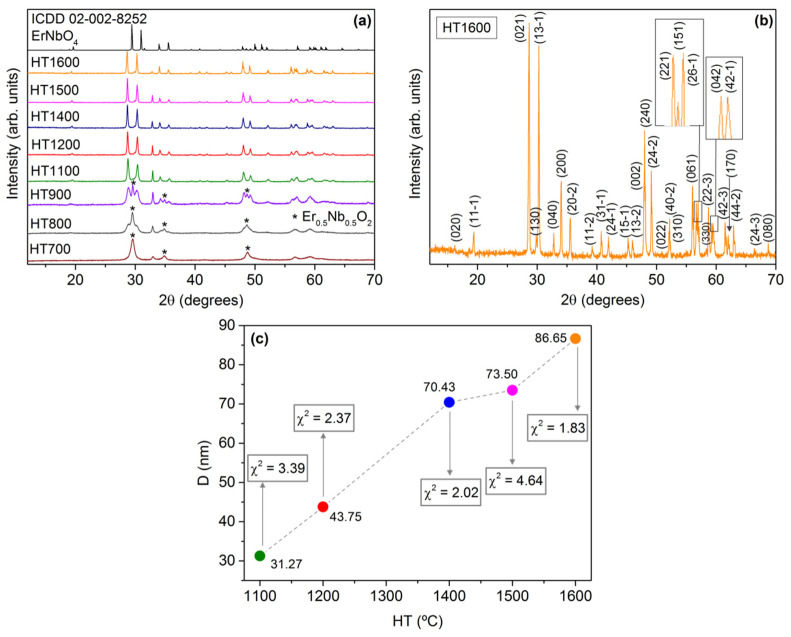
(**a**) X-ray diffraction patterns of the prepared samples; (**b**) measured diffractogram of the sample heat-treated at 1600 °C and Miller indexed planes; (**c**) crystallite size of the samples with pure ErNbO_4_, as a function of heat treatment temperature, estimated through the Rietveld analysis.

The XRD patterns of the prepared samples, shown in Figure 2a, were compared with the data from the standard ICDD codes 04-002-8252 [16], also depicted in the figure, and 04-002-8259 [16]. The formation of the tetragonal Er_0.5_Nb_0.5_O_2_ crystalline phase, detected in the sample HT at 700 °C, is related to the exothermic phenomenon observed in Figure 1. The transformation of Er_0.5_Nb_0.5_O_2_ into the monoclinic ErNbO_4_ crystalline phase can be justified by the endothermic phenomena depicted in the DTA thermogram (Figure 1). The sample HT 1100 shows the monoclinic structure as a single phase. Sharp reflections can be observed in the XRD patterns of the samples, indicating a complete crystallization. Heat treatments above this temperature will not change the crystalline phase. Nevertheless, they will promote an increase in the crystallite size.

Figure 2b shows the diffractogram of sample HT1600 and the corresponding diffracting planes with their Miller (hkl) index assignments estimated by the Rietveld structural refinement software. For the samples with pure ErNbO_4_, the crystallite sizes, D, were also estimated and are depicted in Figure 2c, as well as the fitting parameter $\chi^{2}$, which shows the quality of the Rietveld refinement simulations. As one can see, the crystallite size increases, almost linearly, with the heat treatment temperature.

### 3.2. Morphological Analysis

**Figure 3:**
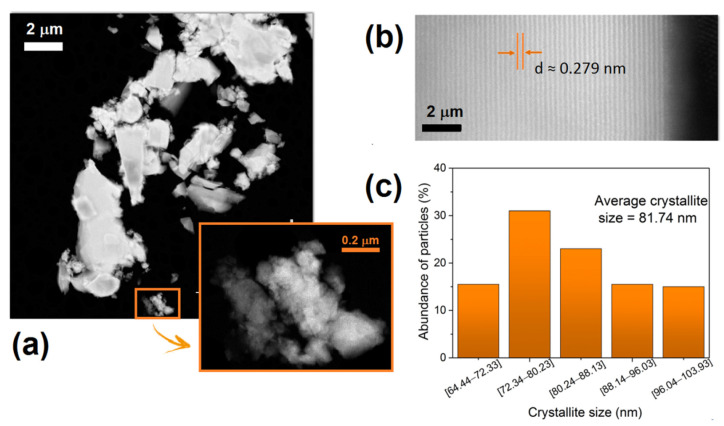
(**a**) STEM micrographs of the HT1600 sample; (**b**) high-resolution STEM micrograph along (040); (**c**) crystallite size distribution and abundance.

Figure 3a displays the STEM dark-field micrograph of the HT1600 sample, where sphere-shaped crystals of ErNbO_4_ can be well seen in the inset. Figure 3b shows a high-resolution STEM micrograph of the same sample, where the lattice fringes are visible. The average inter-fringe was measured to be 0.279 nm, corresponding to the hkl interplanar distance of the (040) planes, in line with the XRD results.

The crystallite size distribution and abundance are presented in Figure 3c. The average crystallite size estimated was 81.74 nm, which agrees with the result obtained from the XRD measurements.

**Figure 4:**
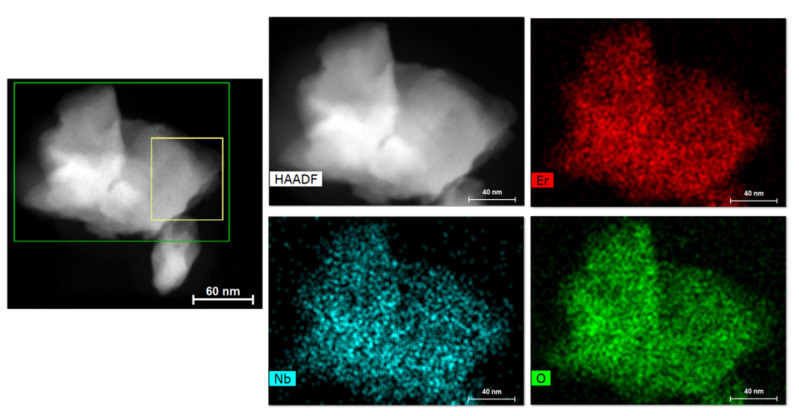
STEM-EDX mapping analysis of the sample HT1600.

Figure 4 shows the STEM-EDX mapping analysis of sample HT1600, where the homogeneous distribution of Er, Nb, and O elements on the surface of the particles can be seen. Furthermore, no other elements were detected as contaminants/impurities.

**Figure 5:**
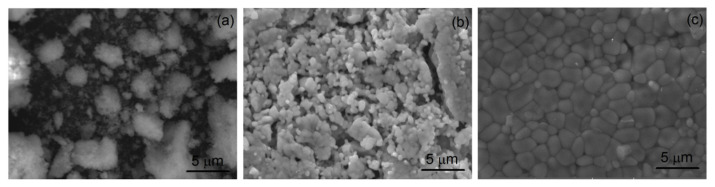
SEM micrographs of the samples: (**a**) HT700; (**b**) HT800; (**c**) HT900.

Figure 5 shows the SEM micrographs of samples HT700, HT800, and HT900, where the Er_0.5_Nb_0.5_O_4_ phase was also identified. The increase in the treatment temperature promotes the grain growth and an increase in the homogeneity of the morphology, with the sample HT900 showing well-defined grain boundaries. 

**Figure 6:**
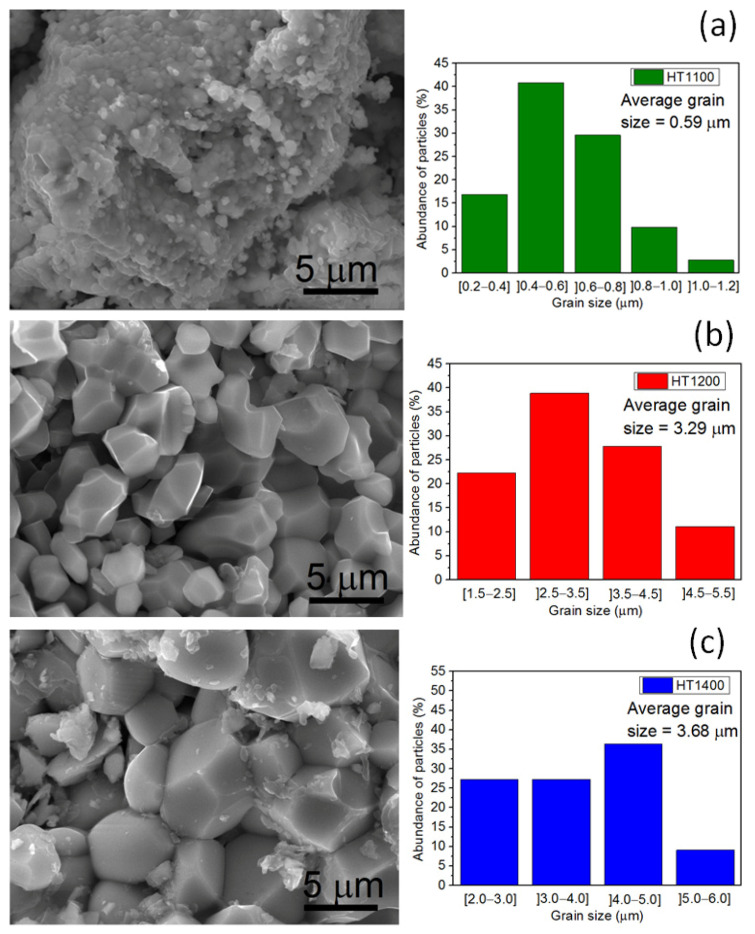

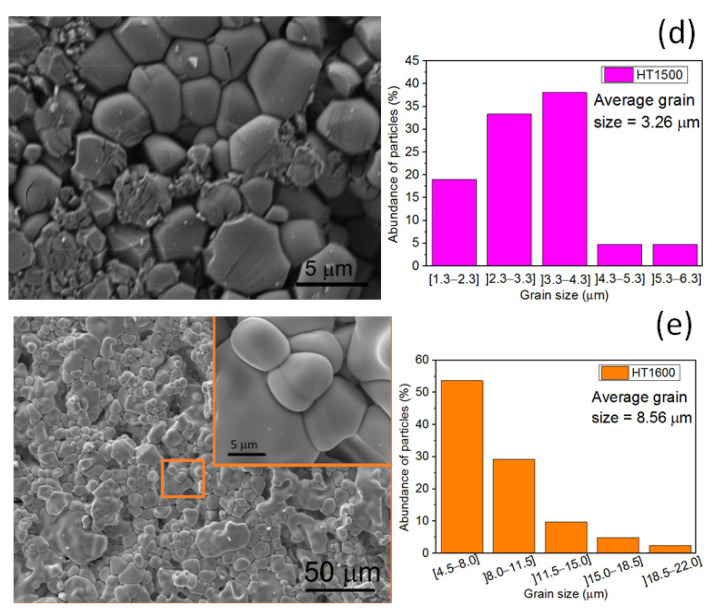
SEM micrographs and grain size distribution graphs of the studied samples: (**a**) HT1100; (**b**) HT1200; (**c**) HT1400; (**d**) HT1500; (**e**) HT1600.

Figure 6 displays the SEM micrographs and the grain size distribution graphs of the samples with the single phase of ErNbO_4_. From Figure 6a, particles with different morphologies and sizes can be seen, with the smaller grains showing a spherical geometry. The grain size of the HT1100 sample is mainly distributed in the range of 0.4–0.8 µm, the average grain size is 0.59 µm and the maximum is about 1.2 µm. For the HT1200 sample depicted in Figure 6b, the grains show a prismatic habit, with the size mainly distributed in the 2.5–4.5 µm range, an average grain size of 3.29 µm and a maximum of about 5.2 µm. The HT1400 and HT1500 samples, presented in Figure 6c,d, show similar features, exhibiting average grain sizes of 3.68 and 3.26 µm, respectively. The HT1600 sample, presented in Figure 6e, exhibits a major grain growth, showing an average grain size of 8.56 µm. Besides, there is evidence of the occurrence of coalescence.

### 3.3. RT Optical Analysis

**Figure 7:**
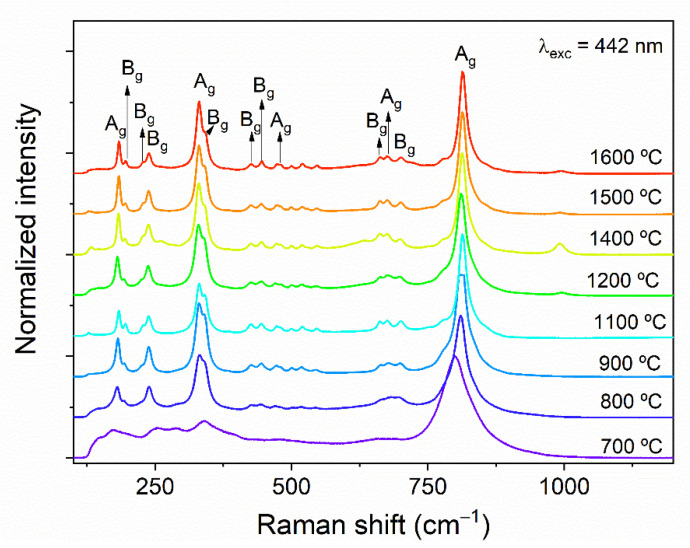
Raman spectra of the erbium niobate samples heat-treated at different temperatures. The spectra were acquired with the 442 nm line of a He-Cd laser in the backscattering configuration. Assignments according to [18].

Raman analysis of all HT samples was assessed and the obtained spectra are depicted in Figure 7. Inspection of the spectrum collected from the sample HT700 reveals that its spectral shape is considerably different from the ones heat-treated at higher temperatures. While in the former case, 7 resonances can be clearly distinguished in the spectrum, for the latter, up to 17 peaks were identified, becoming more pronounced for HT > 900 °C. In fact, according to previous XRD results on similar samples [17], at 700 °C, the samples are dominated by an Er_0.5_Nb_0.5_O_2_ tetragonal phase, which prevails for temperatures lower than 1100 °C. Above such temperature, the XRD patterns were indexed to a single crystalline phase, corresponding to the ErNbO_4_ monoclinic structure. Indeed, RENbO_4_ is known to typically possess a monoclinic crystalline structure, belonging to the space group $C2/c\;={\;C}_{2h}^{6}$, even though they can undergo a reversible phase transformation to a tetragonal structure, with space group $C_{4h}^{6}$, which is only stable at temperatures > 700 °C [3,18]. In the case of the monoclinic structure, the oxygen ions are positioned at the 8f sites, which have C_1_ symmetry, and the cations fill the 4e sites with C_2_ symmetry [3,18,19]. Such configuration results in four structural units and 12 atoms per unit cell [8,18]. According to Siqueira et al. [18], the Raman spectra are similar to all the RENbO_4_ except for RE = Ce and La [3,18]. Considering the point group C_2h_, the reducible representation at the Brillouin zone center can be written as $\Gamma =8A_{g}⊕\;10B_{g}⊕\;\;8A_{u}⊕\;10B_{u}$, which results in a total of 36 phonon modes (including optical and acoustic modes) [8,18]. Among those, the acoustic modes are $A_{u}⊕\;2B_{u}$, with 18 remaining Raman active optical modes, $8A_{g}⊕\;10B_{g}$ [18]. The same authors [18] suggested that the phonon modes appearing at the lowest energies (<300 cm^−1^) are related to the RE units, as their peak position shifts depending on the crystal radii of the RE ion. The differences in the RE radius lead to different packings of the ${\mathrm{NbO}}_{4}^{-3}$ units, which affects the Nb–O distances (decreasing as the RE radii decrease) [8,18]. On the other hand, anti-symmetric Nb–O vibrations are associated with the vibrational modes observed at ~400–500 and ~600–700 cm^−1^, while the most intense peaks at ~300 and 800 cm^−1^ are assigned to the symmetric Nb–O vibrations of the NbO_4_ tetrahedra [8,19,20]. In the present case, and for the samples HT >1100 °C, 13 of the modes identified in the work of Siqueira et al. [18] for the monoclinic structure were also observed and indexed, as shown in Figure 7. It is worth noting that the modes below 180 cm^−1^ could not be identified here, likely due to the filter used to remove the contribution of the Rayleigh scattering during the collection of the Raman signal. Nevertheless, 4 additional small peaks that were not reported by those authors (or others [7,19,21]) are also present in the displayed spectra, specifically at 501, 520, 547, and 777 cm^−1^. The origin of these peaks is not yet clear. Even considering that a residual tetragonal phase was present and not detected by XRD [17], only the shoulder at 777 cm^−1^ is fairly coincident with the expected vibrational modes for such case [19]. 

**Figure 8:**
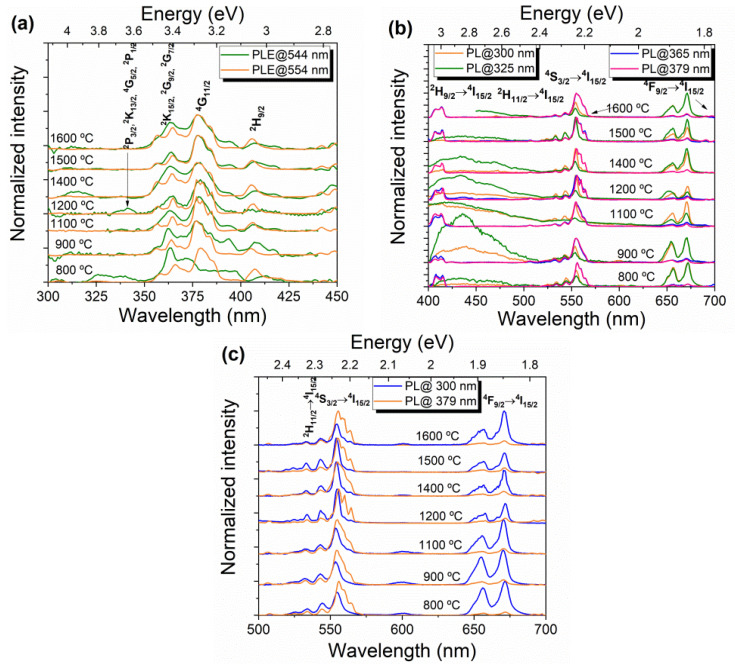
(**a**) RT; (**b**) PLE; (**c**) PL spectra of heat-treated (HT) erbium niobate samples.

Figure 8 displays the RT PLE (Figure 8a) and PL (Figure 8b,c) spectra of the analyzed samples. All samples exhibit the expected intra-4*f*^11^ transitions of the trivalent Er ion, with their typical narrow atomic-like emission lines. Similar features were found for all HT samples both in PLE and PL, independently of the fact that the ones HT at 800 and 900 °C still present a tetragonal Er_0.5_Nb_0.5_O_2_ phase [17], as mentioned above. PLE spectra monitored at the green ^2^H_11/2_, ^4^S_3/2_→^4^I_15/2_ emission (Figure 8a) reveal that the main population of the emitting states arises from the high energetic Er^3+^ multiplets: P_3/2_, ^2^K_13/2_, ^4^G_5/2_, ^2^P_1/2_ at ~343–345 nm, ^2^K_15/2_, ^2^G_9/2_, ^2^G_7/2_ at ~353–372 nm, ^4^G_11/2_ at ~372–390 nm and ^2^H_9/2_ peaked at ~406 nm. Here, 2S+1 accounts for the spin multiplicity, J is the total angular momentum and each ^2S+1^L_J_ term corresponds to a multiplet with 2J+1 states that are split into Stark levels by the action of the local crystal field present at the ions’ site in the crystalline matrix. These results are in agreement with the ones reported by Hirano and Ishikawa [22], as well as Zhang et al. [12,23], obtained from the absorption spectra. Regarding the down-shifted PL spectra (Figure 8b,c), photon excitation with energies higher and resonant with the mentioned electronic excited levels, particularly ^2^K_15/2_, ^2^G_9/2_, ^2^G_7/2_, and ^4^G_11/2_ which peaked at 365 and 379 nm, respectively, leads to the observation of several intraionic PL lines. When excited resonantly with the ion excited energy levels, the PL spectra depict mainly three sets of emission lines: the first in the ultraviolet/blue (400–422 nm), corresponding to the intraionic transitions from the ^2^H_9/2_ multiplet; the second in the green/yellow spectral region (500–580 nm), assigned to transition from ^2^H_11/2_ and ^4^S_3/2_ excited states; and, finally, the third in the red (640–690 nm), resultant from the transitions from the ^4^F_9/2_ to the ^4^I_15/2_ ground state. The present PL features are in line with previous works on ErNbO_4_ samples prepared by different methods [12,19,22,23]. In the present case, both the PLE and PL spectra do not shift significantly with the HT temperature, although some changes in the excitation/emission lines profile are observed, especially for temperatures below 1100 °C. This is due to the presence of the additional tetragonal Er_0.5_Nb_0.5_O_2_ phase, which results in a different symmetry environment for the Er ions incorporated in that phase. 

The intensity ratio between the green/yellow ^2^H_11/2_,^4^S_3/2_→^4^I_15/2_ and red ^4^F_9/2_→^4^I_15/2_ lines was found to be strongly sensitive to the excitation energy and follows the same tendency in all studied samples: (i) by exciting the samples with photon energies resonantly into ^4^G_11/2_, the intensity of the green/yellow transition is stronger than the red one; (ii) for higher photon energy excitation, the intensity of the red emission increases relatively to the green/yellow one. The latter excitation condition also favors the presence of a broad emission band that may be due to intrinsic defects. The spreading in energies of such emission band can lead to Er^3+^ re-absorption, resulting in a different intensity ratio of the green/red emission bands. However, as the phenomenon is observed in both crystalline hosts, a more likely explanation for the identified behavior is to consider that efficient nearby ion–ion interactions are promoted in the erbium niobate hosts. It is well established that the green/yellow to red emission tuning can be accomplished by increasing the dopant concentration in oxide hosts, which is explained by nonradiative energy transfer, including cross-relaxation mechanisms, multiphonon deexcitation, and other nonradiative processes [24,25,26,27]. Cross-relaxation processes can be used to selectively tune the Er^3+^ luminescence by enhancing the emission from one excited level while quenching it from another. In particular, the depletion of ^2^H_11/2_, ^4^S_3/2_ states to the ^4^F_9/2_ level by multiphonon deexcitation and cross-relaxation processes such as the ones involving the long-lived ^4^I_13/2_ and ^4^I_11/2_ multiplets (which in turn repopulate the ^4^F_9/2_ level) is known to account for the tuning of the emission color from green to red in several oxide hosts [24,25,26,27], as shown in Figure 8c for the here studied erbium niobate samples. Therefore, as the color tuning arises from the distinct excitation energies, it is fair to assume that the feeding of the ^4^F_9/2_ multiplet by the cross-relaxation mechanism is favored when the excitation is performed by higher photon energies.

**Figure 9:**
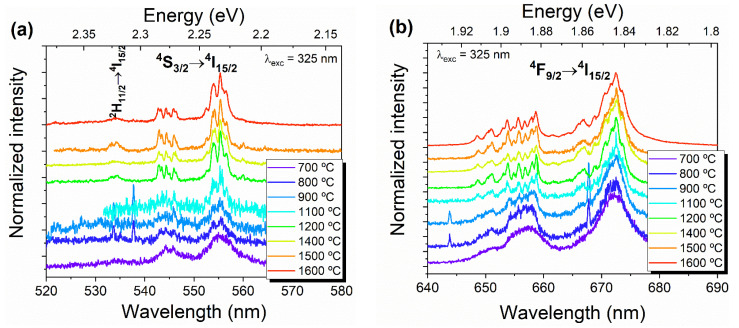

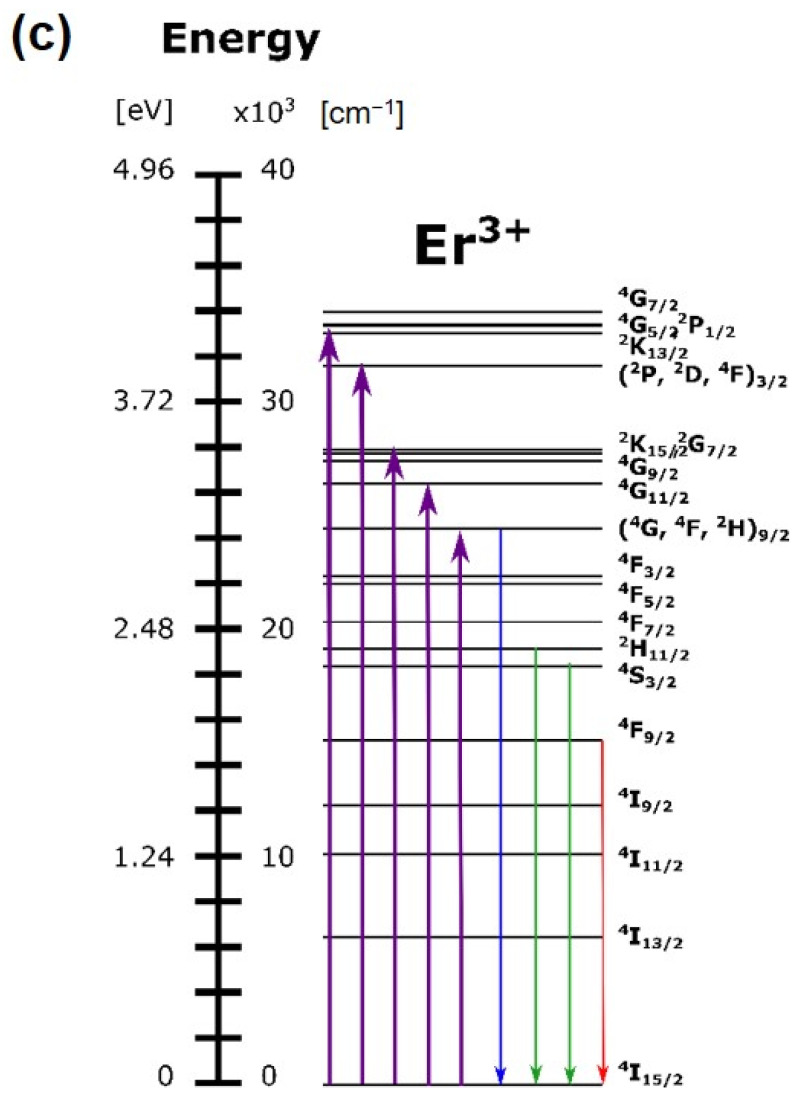
(**a**) High-resolution RT normalized; (**b**) PL spectra of HT erbium niobate samples assessed with the 325 nm line of a cw He-Cd laser; (**c**) schematic free ion energy levels of Er^3+^ and observed transitions in erbium niobate (energy level constructed based on [13]).

Figure 8c displays a more detailed look at the transitions between the ^2S+1^L_J_ multiplets, in the spectral range from 500 to 700 nm. The number of splittings depends on the local field symmetry, with a maximum number of 2J+1 for the J integer and J+1/2 for the J half-integer, as is the case of Er^3+^ [28,29]. For a monoclinic structure, the RE ion is typically located at a C_2_ symmetry site, as referred to above in the Raman discussion, which corresponds to a low-symmetry environment. As such, a full splitting of (2J+1)/2 of the ^2S+1^L_J_ multiplets is expected. Since for the Er^3+^ ion, J is the half-integer, each level should be double degenerated [19]. To gain a better insight on the number of Stark levels that we could identify in these samples, high-resolution spectra were acquired by exciting the samples with a 325 nm laser line, which corresponds to photon energies higher than the ^2^G_9/2_, ^2^K_15/2_, ^2^G_7/2_ multiplets. The recorded spectra are represented in Figure 9a,b for the green/yellow ^2^H_11/2_,^4^S_3/2_→^4^I_15/2_ and red ^4^F_9/2_→^4^I_15/2_ Er^3+^ transitions, respectively. The ^2^H_9/2_→^4^I_15/2_ transition was not assessed as, under such excitation conditions, the violet/blue spectral region is dominated by a broad emission band (see Figure 8b). A schematic representation of the free ion energy levels diagram of Er^3+^ is illustrated in Figure 9c, showing the intraionic electronic transitions that were observed in the here reported samples and the respective assignments. 

For the samples HT at temperatures >900 °C, several narrow lines for green/yellow and red transitions are well discerned, as anticipated taking into account that the Er ions are expected to be in low-symmetry sites [3,19,24]. Hence, the ^4^S_3/2_ and ^4^I_15/2_ levels should split into a maximum of two and eight Stark levels, respectively, giving rise to 2 × 8 = 16 transitions between these levels, considering only the contribution of one optical center. In the case of ^4^F_9/2_, a maximum of five Stark levels are expected, and thus 5 × 8 = 40 emission lines for the red ^4^F_9/2_→^4^I_15/2_ transitions [19,23]. The peak positions identified for each of the mentioned Er^3+^ transition lines for the HT samples at temperatures >900 °C are listed in Table 1. In the case of the ^4^S_3/2_→^4^I_15/2_ transition, we could identify between 8 (HT 1100 °C) and 14 (HT 1200 °C) lines, while for the ^4^F_9/2_→^4^I_15/2_ transition, up to 24 (HT 1400 °C) were observed. The peak position of these lines is in good agreement with the ones reported by Zhang [23]. The fact that not all the Stark levels can be identified in the present case is likely related to the overlap of some of the emitting lines at RT.

Regarding the samples treated at <900 °C, the broad feature observed for the intraionic transitions is likely related to the presence of the two phases, monoclinic ErNbO_4_ and tetragonal Er_0.5_Nb_0.5_O_2_, as stated above, thus exhibiting the ions in more than one symmetry environment than the C_2_ one, which results in an overlap of several emission lines. Hence, this information was not included in Table 1.

**Figure 10:**
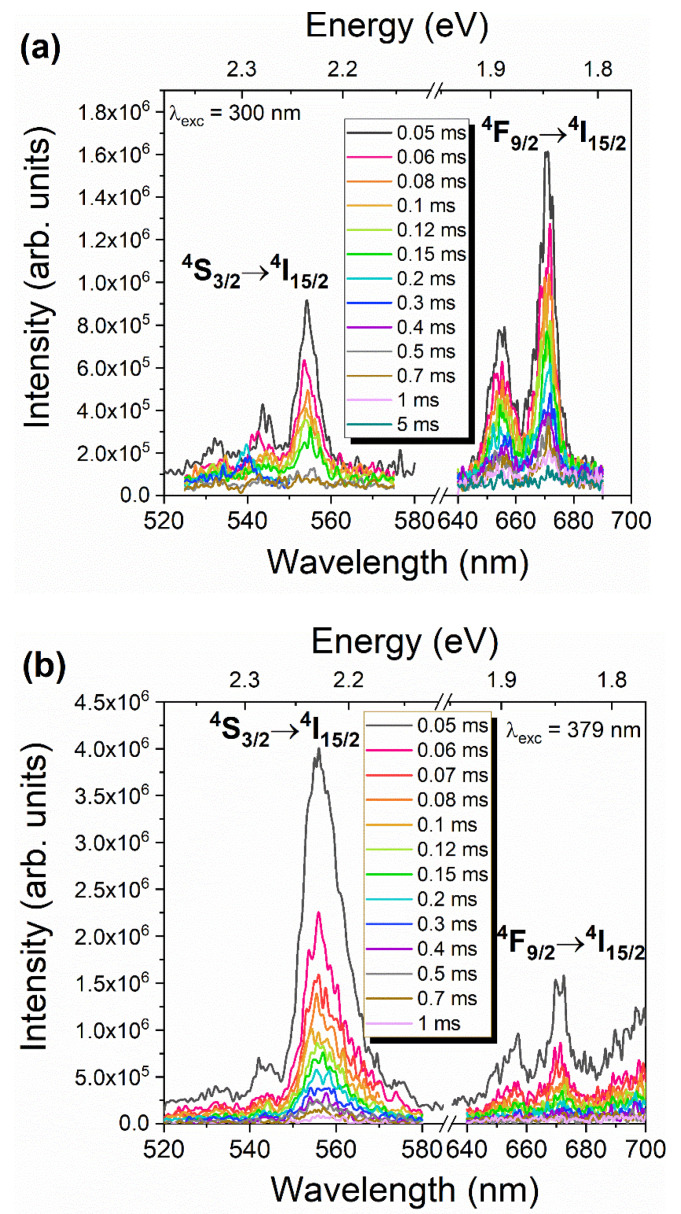
TRPL spectra obtained for the HT1200 sample under two excitations: (**a**) 300 nm; (**b**) 379 nm.

Following our previous discussion, differences in the recombination dynamics of the ^4^F_9/2_→^4^I_15/2_ transition are expected to be observed by pumping the samples with distinct photon energies. Figure 10 shows the representative TRPL spectra acquired for the monoclinic ErNbO_4_ samples under two excitations, 300 nm (which promotes the enhancement of the red ^4^F_9/2_→^4^I_15/2_ transition) and 379 nm (resonant with the ^4^G_11/2_ multiplet). Time delays between 0.05 and 5 ms were employed using a fixed sample window of 0.2 ms. Independently of the used excitation, the intensity of the emission lines associated with the ^2^H_11/2_,^4^S_3/2_→^4^I_15/2_ transitions completely vanished for delays higher than 0.7 ms, disappearing after 1 ms. These data indicate that similar ^2^H_11/2_,^4^S_3/2_→^4^I_15/2_ decay is measured for both excitation conditions, which is in the range of hundreds of µs. In the case of the red ^4^F_9/2_ →^4^I_15/2_ transition, it is seen that under 379 nm excitation, the emission is only distinguishable for delays <0.15 ms, while when it is excited with 300 nm, this evidences a lower reduction in the magnitude of its intensity for shorter delays, decreasing by one order of magnitude only for times equal or higher than 1 ms. This behavior suggests that a rise time on the dynamics of the red ^4^F_9/2_→^4^I_15/2_ recombination process occurs, in line with the above-discussed multiplet feeding by energy transfer processes.

## 4. Conclusions

In this work, the RT optical properties of erbium niobate micro- and nanoparticles prepared by the citrate sol–gel method and subsequently heat-treated at temperatures in the interval from 700 to 1600 °C were reported. Differential thermal analysis and thermogravimetric measurements reveal that the tetragonal Er_0.5_Nb_0.5_O_2_ crystalline phase starts to form at temperatures of 670 °C, whereas for temperatures higher than 800 °C, the monoclinic ErNbO_4_ crystalline phase develops, as proven by X-ray diffraction. For heat treatment temperatures above 1100 °C, the monoclinic ErNbO_4_ crystalline phase prevails. The crystallite size of the latter was found to increase linearly with increasing heat treatment temperatures, reaching a maximum value of 87 nm for the HT1600 sample, which evidences a sphere-shaped morphology. For the case where the two crystalline phases are present (700 to 900 °C), a noticeable grain growth accompanied by an increase in the morphology homogeneity with well-defined grain boundaries was identified by electronic microscopy. Additionally, samples with the monoclinic ErNbO_4_ crystalline phase (HT from 1100 to 1600 °C) evidence an increase in the average grain sizes from a few microns to 8.56 µm for the highest treatment temperature, accompanied by a modification of the grain morphology from spherical to prismatic. Grain coalescence is observed for the highest temperature. RT Raman spectroscopy corroborates the presence of the two crystalline phases for heat treatments below 900 °C and a single monoclinic phase for higher temperatures. For the latter, thirteen of the eighteen optical vibrational modes were observed and indexed. RT spectroscopic studies reveal that Er^3+^ optical activation was achieved in all samples. The photoluminescence spectra show the main visible recombination of the green/yellow ^2^H_11/2_,^4^S_3/2_→^4^I_15/2_ and red ^4^F_9/2_→^4^I_15/2_ intraionic transitions, which are preferentially populated via Er^3+^ high-energy excited multiplets. Independently of the erbium niobate crystalline structure, the intensity ratio between the green/yellow and red intraionic transitions was found to be strongly sensitive to the excitation energy. After pumping the samples with a resonant excitation into the ^4^G_11/2_ excited multiplet, a green/yellow transition stronger than the red one was observed, whereas the reverse occurred for higher excitation photon energies. As confirmed by time-resolved photoluminescence, the observed behavior was discussed considering cross-relaxation mechanisms involving intermediate long-lived Er^3+^ levels that repopulate the red-emitting multiplet. Thus, a controllable selective excited tunable green to red color in erbium niobate samples was achieved.

## Figures and Tables

**Table 1:** Assignments and peak position of the Er^3+^ intrashell lines for the ErNbO_4_ samples heat-treated at temperatures >900 °C.

Peak Position of the Transition Lines (±0.1 nm)	HT Temperature (°C)				
	1100	1200	1400	1500	1600
^2^H_11/2_→^4^I_15/2_		533.2 534.3		533.2 534.1	
^4^S_3/2_→^4^I_15/2_	544.4 545.9 552.2 553.9 555.3 555.7 556.6 557.4	542.9 543.4 544.3 545.1 545.1 545.9 546.9 552.5 554.0 555.3 556.5 558.3 559.3 559.9	542.9 543.4 544.3 545.1 545.8 546.3 552.6 553.8 554.3 555.3 556.0 556.6 559.9	542.9 543.5 544.4 545.0 545.9 546.5 552.5 553.8 554.1 555.4 555.9 556.6 560.1	542.7 543.3 544.3 544.9 545.9 546.4 552.5 554.0 554.3 555.3 555.9 556.5
^4^F_9/2_→^4^I_15/2_	648.8 650.4 651.1 652.9 653.7 654.7 655.7 656.8 657.9 658.7 661.6 666.5 668.7 670.5 672.5 673.6	648.6 650.2 651.0 653.0 653.8 654.8 655.1 655.7 656.8 658.0 658.7 661.7 665.9 667.0 668.7 670.1 672.4 673.5	648.6 650.3 651.0 653.1 653.8 654.9 655.7 656.8 657.7 658.1 658.7 661.8 663.5 664.3 665.9 666.5 667.0 667.8 668.7 670.7 671.7 672.6 673.6	648.8 650.3 651.1 652.4 652.9 653.8 654.8 655.7 656.8 657.9 658.7 666.8 668.8 670.5 671.8 672.5 673.6	648.5 650.3 651.0 653.0 653.7 654.8 655.6 656.7 657.9 658.7 661.6 663.3 664.3 665.9 666.8 668.8 670.5 671.6 672.5 673.5

## Data Availability

not applicable.
